# A funfair without the candy floss: engaging communities to prevent diabetes in Nepal

**DOI:** 10.1016/j.puhe.2021.01.012

**Published:** 2021-04

**Authors:** J. Morrison, A. Arjyal

**Affiliations:** a University College London, Institute of Global Health, London, UK; b Herd International, Thapathali, Kathmandu, Nepal

**Keywords:** Community engagement, Art, Participatory action research, Type 2 diabetes, Non-communicable diseases

## Abstract

**Objectives:**

The World Health Organization estimates that 422 million people have diabetes, three-quarters of whom live in low- and middle-income countries. Global action plans to address non-communicable diseases (NCDs) recognise the centrality of community engagement to create an enabling environment within which to address risk factors.

**Study design:**

In this article, we describe and critically reflect on a cocreated community engagement approach to address type 2 diabetes in the southern plains of Nepal. We coproduced the engagement approach with 40 artists from the Janakpur Women's Development Centre to create an environment for dialogue about diabetes and NCD risk between artists and the general public.

**Methods:**

We used participatory action research to produce contextually relevant interactive methods and materials. Methods included artists' peer research to inform creative workshops, a drama performed in 19 villages and a two-day funfair in a public park. We used qualitative and participatory methods to analyse the effect of this engagement and reflect on lessons learned.

**Results:**

Around 2000 people saw the drama, and around 4000 people attended the funfair. Community dialogue about prevention of diabetes was facilitated by drama and through games and songs at the funfair. Artists grew confident to interact with their peers and drama audiences about the causes of diabetes and prevention strategies. Despite crowds at the funfair, it was difficult to reach women because the venue was often used by men and boys, and patriarchal norms prevent women from free movement. Village interactions were able to engage a more mixed audience.

**Conclusion:**

Innovative, asset-based community engagement about diabetes and other NCDs at scale is possible through locating, building on and strengthening community resources to address local health issues. Engagement could be enhanced by considering the gendered nature of community engagement spaces and by increasing opportunities for interaction between artists and the general public through more intimate and large-scale events.

## Highlights

• There is a need for innovative approaches to engage with communities to prevent and control non-communicable diseases.
• We coproduced a population-based community engagement approach with artists in southern Nepal using peer research, a community drama and a two-day funfair in a public park.
• We successfully engaged with more than 4000 people who enjoyed our creative art-based approach, which stimulated dialogue about diabetes among artists and the general public.
• Innovative, asset-based community engagement at scale is possible through locating, building on and strengthening community resources to address local health issues, creating empowered communities.
• Engagement could be enhanced by considering the gendered nature of public engagement spaces and by increasing opportunities for interaction between artists and the general public through more intimate and large-scale events.

Three-quarters of the 422 million people who have diabetes live in low- and middle-income countries.^1^ In South Asia alone, an estimated 96 million people have diabetes. Ninety percent of these people have type 2 diabetes, a largely preventable disease.^2^ There is an urgent need for multisectoral approaches to create enabling environments to address risk factors and ensure that community engagement is well integrated in non-communicable disease policies and plans.^3^ Many multisectoral action plans approach community engagement through mass media,^4^ despite limited evidence of its effectiveness in changing behaviours.^5^ Although it may create an enabling environment, mass media provides inadequate opportunity for interaction to develop social support networks, which have been key to the success of peer and group-based interventions among high-risk populations.^6^ Research from rural Bangladesh found that a participatory community group-based intervention reduced the combined prevalence of type 2 diabetes and hyperglycaemia by 21% in intervention areas when compared with control areas.^7^ The interactive nature of the intervention was integral to its success.^8^ There is a need to further innovate and develop effective community engagement interventions to address diabetes. We describe such an intervention in Nepal and reflect on lessons learned to inform future initiatives.

We worked with 40 artists from the Janakpur Women's Development Centre in the southern plains of Nepal to cocreate a population-based community engagement approach for diabetes prevention. We sought to build on local assets of an established female community art centre and a strong tradition of Mithila art to engage communities about diabetes. Mithila art is traditionally painted on the outside of houses by women. A diverse and resilient group of artists work at the centre, many of whom have been socially and economically marginalised by illiteracy, unstable home environments, chronic illness, disability and widowhood. An asset-based approach seeks to use and build on local collective skills, resources, talents and relationships to improve health and well-being.

**Fig. 1:**
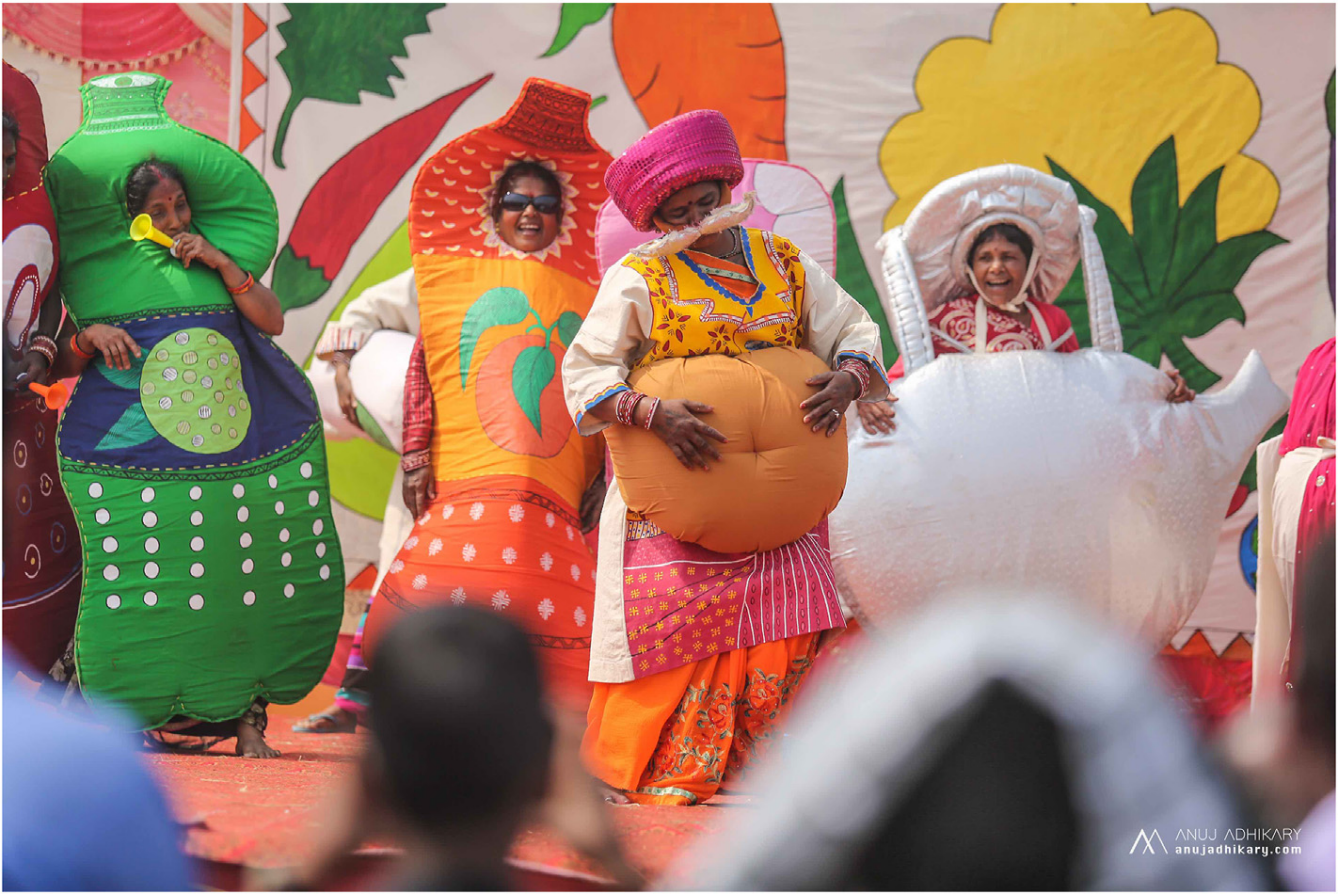
Artists perform a drama about how to prevent diabetes.

We used participatory action research,^9^ collaboratively and iteratively designed the engagement based on context-specific artistic forms of expression and focused on local issues. To enable this, artists undertook 16 peer interviews to explore local experiences and understandings of diabetes. Initially, artists lacked confidence, and we developed this through training, practice, positive reinforcement and participatory development of visual tools (https://www.ucl.ac.uk/global-health/sites/global-health/files/pictorial_consent_process_final.pdf). We found that diabetes was poorly understood and beliefs about it being a communicable disease caused stress and anxiety among those with diabetes and their families, perpetuating social stigma. The cost of diabetes care was prohibitively high, which often caused guilt about the impact of diabetes on the family. Given the lack of specialist health care outside the capital Kathmandu, we agreed that a focus on prevention was a priority for engagement. A local health worker helped us to disentangle truths and untruths, and we designed art and games for a two-day funfair and created a travelling drama to promote active learning and stimulate conversations about diabetes (Fig. 1) (https://www.youtube.com/watch?v=8orIX40-ILw). Artists performed the drama in 19 villages and markets with audiences of around 100 people and hosted a two-day funfair in a public park in the urban town of Janakpur. Around 4000 people attended the funfair, 800 of whom had free blood glucose testing. We evaluated our community engagement through three focus group discussions (FGDs) with artists, two FGDs with women who had attended the funfair, one FGD with men who had attended and six artist peer interviews with those who had attended the funfair. Artists and researchers also engaged in critical self-reflection, making notes about what worked well and what worked less well throughout the engagement process.

At the funfair, giant snakes and ladders, games to knock over ‘unhealthy’ objects and games to feed ‘unhealthy’ figures ‘healthy’ food were popular among men and children, and women preferred the Zumba exercise routine. Stage shows of the drama and songs were more popular than the immersion tunnel and head-in-the-hole photo stands. Artists' confidence grew throughout the project, and they felt able to teach others about diabetes and to perform on stage. The travelling drama attracted large crowds in market areas, with many more men than in rural villages, where audiences were smaller and with a mix of men, women and children. Village dramas engaged the old and young, and the smaller audiences made it easier to initiate dialogue after the drama. Some community drama audiences felt that artists were acting inappropriately and feared their behaviour would erode social norms. An artist told us, “People were saying: ‘These women have come to destroy our village.’” But as a group, their fear of social shame was less, and interactions with crowds afterwards led to increased understanding about why the women were performing and were effective in promoting dialogue about how to prevent and control diabetes.

Despite crowds at the funfair, we found it more difficult to reach women because the venue was not a place where women or families would usually go. Gender norms are patriarchal, and it is not considered decent for women to be outside of the home without purpose. Our mixed gender advisory committee recommended the funfair location—a large public park where men and boys often play cricket—but without adequate consideration of the gendered nature of the space. Conventional funfairs move from place to place. Taking the drama and the funfair around villages and more intimate locations such as schools or hospitals could enable equal access to participate among men and women and facilitate more interaction between artists and the public.

Innovative, asset-based community engagement at scale is possible through locating and strengthening community resources to address local health issues. Asset-based approaches have been critiqued for seeking to shift the focus away from structural inequalities driving poor health,^10^ and we acknowledge that combining community engagement with health system strengthening to deal with the increased demand for services is important for such initiatives.

Finally, evaluating community engagement is challenging. Some have advised the capture of contribution as opposed to seeking causal attribution, whereas others have suggested realist approaches to consider complexity.^11^ If the engagement is long-lasting and embedded, measurement of behavioural and biological outcomes is possible, for example, through cluster randomised controlled trial designs, wherein communities are randomly allocated to participate in the engagement process or to serve as control communities. Knowledge, practice and health outcomes such as prevalence of type 2 diabetes and intermediate hyperglycaemia could be measured and compared between trial arms.^7^ But it is important to develop a robust theory of change to maximise the chance of success of the intervention. It is also important to take a pluralist approach to evaluation, using methods that acknowledge how success is viewed by the diversity of implementers and participants.^12^

## Author statements

### Acknowledgements

The authors would like to thank all the artists at the Janakpur Women's Development Centre and all those who participated in and assisted with the public engagement intervention including Ms. Clare Burkert, Ms. Susie Vickery, Mr. Satish Sah, Ms. Awantika Priyadarshani, Ms. Jyoti Mandal, Mr. Dinesh Deokota, our advisory committee members, Prof. David Osrin, Prof. Audrey Prost, Prof. Nish Chaturvedi, Amit and Ruby Shah from SEIT Nepal, Suraj Thakur and his team at Beats and Step Dance School, Pramesh Jha from Minap, Ms. Nani Shova Shakya, Ms. Renu Yadav and Mr. Umesh Yadav from the Nepal Dietician Association, Ms. Roopshree Joshi from World Education and photographer Mr. Anuj Adhikary.

### Ethical approval

This study received ethical approval from the Nepal Health Research Council 235/2018 and from the UCL Ethics Committee 4199/005.

### Funding

This work was funded by a 10.13039/100010269Wellcome Trust Public Engagement Award (209722/Z/17/Z).

### Competing interests

The authors have no competing interests to declare.
